# Prevalence of intronic repeat expansions in *RFC1* in Dutch patients with CANVAS and adult-onset ataxia

**DOI:** 10.1007/s00415-022-11275-9

**Published:** 2022-07-21

**Authors:** Fatemeh Ghorbani, Jelkje de Boer-Bergsma, Corien C. Verschuuren-Bemelmans, Maartje Pennings, Eddy N. de Boer, Berry Kremer, Els K. Vanhoutte, Jeroen J. de Vries, Raymond van de Berg, Erik-Jan Kamsteeg, Cleo C. van Diemen, Helga Westers, Bart P. van de Warrenburg, Dineke S. Verbeek

**Affiliations:** 1 Department of Genetics, HPC CB50, University Medical Center Groningen, University of Groningen, P.O. Box 30001, 9700 RB Groningen, The Netherlands; 2 Department of Human Genetics, Radboud University Medical Center, Nijmegen, The Netherlands; 3 Department of Neurology, University Medical Center Groningen, University of Groningen, Groningen, The Netherlands; 4 Department of Clinical Genetics, Maastricht University Medical Center, Maastricht, The Netherlands; 5 Department of Otorhinolaryngology and Head and Neck Surgery, School for Mental Health and Neuroscience, Maastricht University Medical Center, Maastricht, The Netherlands; 6 Department of Neurology, Donders Institute for Brain, Cognition, and Behaviour, Radboud University Medical Center, Nijmegen, The Netherlands; 7 Expertise Center Movement Disorders Groningen, University Medical Center Groningen, Groningen, The Netherlands

**Keywords:** CANVAS, Adult-onset ataxia, Repeat expansion, *RFC1*, Optical genome mapping

## Abstract

Recently, an intronic biallelic (AAGGG)_n_ repeat expansion in *RFC1* was shown to be a cause of CANVAS and adult-onset ataxia in multiple populations. As the prevalence of the *RFC1* repeat expansion in Dutch cases was unknown, we retrospectively tested 9 putative CANVAS cases and two independent cohorts (A and B) of 395 and 222 adult-onset ataxia cases, respectively, using the previously published protocol and, for the first time optical genome mapping to determine the size of the expanded *RFC1* repeat. We identified the biallelic (AAGGG)_n_ repeat expansion in 5/9 (55%) putative CANVAS patients and in 10/617 (1.6%; cohorts A + B) adult-onset ataxia patients. In addition to the AAGGG repeat motif, we observed a putative GAAGG repeat motif in the repeat expansion with unknown significance in two adult-onset ataxia patients. All the expanded (AAGGG)_n_ repeats identified were in the range of 800–1299 repeat units. The intronic biallelic *RFC1* repeat expansion thus explains a number of the Dutch adult-onset ataxia cases that display the main clinical features of CANVAS, and particularly when ataxia is combined with neuropathy. The yield of screening for *RFC1* expansions in unselected cohorts is relatively low. To increase the current diagnostic yield in ataxia patients, we suggest adding *RFC1* screening to the genetic diagnostic workflow by using advanced techniques that attain long fragments.

**Supplementary Information:**

The online version contains supplementary material available at 10.1007/s00415-022-11275-9.

## Introduction

The cerebellar ataxias are a group of genetically heterogenous neurodegenerative disorders, characterized by atrophy of the cerebellum that leads to the inability to control balance and coordination [1]. Even though there are many known cerebellar ataxia–causing genes and variations, some adult patients with adult-onset cerebellar ataxia remain genetically undiagnosed [2], and studies to identify the genetic cause are ongoing. It has also been shown that patients can experience a range of clinical symptoms, from pure cerebellar ataxia to more complex clinical phenotypes including for example peripheral neuropathy [3]. One example here is CANVAS, a syndrome of adult-onset, slowly progressive ataxia associated with bilateral vestibulopathy, neuropathy, chronic cough, and autonomic dysfunction [4]. Recently, Cortese et al. reported a biallelic intronic repeat expansion in the gene encoding the replication factor C subunit 1 (*RFC1*) as a cause of CANVAS. They identified four different repeat motifs at the *RFC1* locus: the (AAAAG)_11_, (AAAAG)_n_ and (AAAGG)_n_ repeats, which are considered benign, and the pathogenic (AAGGG)_n_ repeat that causes CANVAS when it occurs in a biallelic state. Interestingly, the biallelic (AAGGG)_n_ repeat expansion also explained quite a number of late-onset ataxia cases. Thus, this expansion can also be considered as a novel cause of late-onset ataxia.

Following these results, various studies have screened regional CANVAS and adult-onset ataxia cohorts for the biallelic (AAGGG)_n_ repeat expansion [5–15] (Supplementary Table 1). In all these studies, the frequency of the biallelic (AAGGG)_n_ repeat expansion was higher in CANVAS cohorts compared to late-onset ataxia cohorts. Additionally, novel mono-allelic repeat motifs such as (AAGAG)_n_ and (AGAGG)_n_ were identified in combination with the mono-allelic (AAGGG)_n_ repeat [5]. Moreover, novel biallelic (ACAGG)_n_ repeats were also found at the *RFC1* locus [7], but whether these previously unreported repeat motifs are the cause of the disease remains to be investigated.

In this study, we aimed to study the prevalence of the biallelic (AAGGG)_n_ repeat expansion in nine cases with clinically putative CANVAS and two cohorts of combined 617 adult-onset ataxia cases from the Netherlands. To determine if the patients carry the biallelic (AAGGG)_n_ repeat expansion, we performed *RFC1-*flanking PCR, repeat primed PCR (RP–PCR), long-range PCR for Sanger sequencing and optical genome mapping to measure the size of the repeat expansion.

## Material and methods

### Patient selection and characterization

Nine patients were clinically suspected of having CANVAS with the presence of adult-onset cerebellar ataxia (age at onset > 25 years), and either sensory axonal neuropathy or vestibulopathy. These nine cases were previously collected as a small series from 2015 to 2020 via the Departments of Neurology and Genetics of the University Medical Center Groningen (UMCG), the Netherlands and the ENT Department of Maastricht University Medical Center, the Netherlands.

We also included two independent cohorts of cases who were not preselected for particular clinical features related to CANVAS or *RFC1*-disease other than the presence of adult-onset ataxia. The first cohort (A) comprised of 395 adult-onset ataxia patients, excluding those with current age < 25 years, who were referred to the Department of Genetics of the UMCG for cerebellar ataxia molecular diagnostics from years 1997 to 2017. These cases remained negative after screening for repeat expansions in the SCA1, 2, 3, 6, 7 and 17 genes and conventional variants in known dominant late-onset ataxia (SCA) genes. The second cohort (B) comprised of 222 ataxia patients with a negative dominant family history and excluding those with current age < 30 years, who had been referred to the diagnostic lab of the Human Genetics department of the Radboud University Medical Center for cerebellar ataxia diagnosis between years 2012–2018. These patients remained genetically undiagnosed after testing for either combined SCA1, 2, 3, 6, 7, and 17 analysis or for exome sequencing. The clinical and paraclinical information for patients in cohorts A and B, carrying (AAGGG)_n_ *RFC1* repeat expansions, was retrospectively collected from the patient reports. Unfortunately, the required information on all *RFC1*-related disease elements was not available and/or not retrievable for all cases, as these in part concerned diagnostic requests from other centers, making it difficult to access patient reports. In most of the cases in cohorts A and B with *RFC1* repeat expansions and bilateral vestibular impairment the history and/or neurologic investigation have been suggestive for vestibulopathy, without specifications on the type of vestibulopathy and how this was established. MRI scans were available for all *RFC1-*positive cases of the putative CANVAS series and for eight *RFC1-*positive cases from the adult-onset ataxia cohorts. The total number of cases who underwent an MRI scan remains unknown. For this study, no additional informed consent was requested as the test is in line with the original request for diagnostic testing.

### Flanking PCR, RP–PCR, long-range PCR and Sanger sequencing

Flanking PCR was performed on the *RFC1* repeat region to identify cases with non-amplifiable products. To prevent false positives, PCR products of the *DCHS1* gene (cohort A) or the *AMELX/Y* genes (cohort B) were simultaneously generated within the same tube/well to serve as an internal control. Supplementary Table 2 lists the primers used for the amplification of the *RFC1, DCHS1* and *AMELX/Y* loci.

**Fig. 1:**
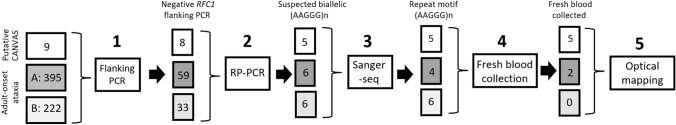
Diagram showing the workflow and the number of patients from each cohort at the start of the study to identify patients with *RFC1* (AAGGG)_n_ expanded alleles. The study started with 3 cohorts including a putative CANVAS cohort consisting of nine cases, and cohorts A and B with 395 and 222 adult-onset ataxia cases, respectively. The subsequent steps included: (1) *RFC1*-flanking PCR to identify cases with a non-amplifiable *RFC1* region, (2) repeat primed-PCR (RP–PCR) to identify patients suspected of carrying biallelic (AAGGG)_n_  expansions, (3) Sanger sequencing (Sanger-Seq) to read the repeat motifs and (4) collect fresh blood and (5) optical genome mapping to determine the size of the repeat expansion

RP–PCR was performed as described by Cortese et al. [4] on the genomic DNA of patients who showed a PCR product for *DCHS1* or *AMELX/Y* but not *RFC1*. The primers used for the RP–PCR specifically targeted three different repeat sequences: I. expanded benign allele (AAAAG)_15–200_, II. expanded benign allele (AAAGG)_40–1000_ and III. expanded pathogenic allele (AAGGG)_400–1000_. The primers, PCR conditions and program used for the RP–PCR can be found in Supplementary Tables 3–5. An ABI 3730xl Genetic Analyzer (Applied Biosystems, Waltham, Massachusetts, USA) was used to analyze the RP–PCR fragment lengths. The resulting data was analyzed using Genemapper software v5 (Applied Biosystems). Based on the RP–PCR, samples that seemed biallelic for the (AAGGG)_n_ repeat expansion were subjected to long-range PCR using the Phusion Flash High-Fidelity PCR Master Mix (Thermo Fisher Scientific, Waltham, Massachusetts, USA). For primers, PCR conditions and program see Supplementary Tables 2, 6 and 7, respectively. Sanger sequencing was performed on the PCR products to analyze the sequence motif of the repeats. The start of the repeat motif is based on a consensus reference sequence preceding the repeat motif. Figure 1 shows the complete workflow.

### Optical genome mapping

Optical genome mapping (Bionano Genomics, San Diego, CA, USA) was used to determine the size of the repeat expansion in *RFC1*. Fresh EDTA blood samples were drawn from patients and immediately frozen at − 80 °C. The DNA isolation, labelling and optical mapping procedure was performed by the Bionano Services Lab (Clermont-Ferrand, France). For each sample, a minimum of 650 µl frozen blood was used to purify ultra-high molecular weight gDNA, following the Bionano Prep SP Frozen Human Blood DNA Isolation Protocol (Bionano Genomics). DNA molecules were labelled using the Direct Label and Stain DNA Labelling Kit (Bionano Genomics). Labelled gDNA samples were loaded on Saphyr chips using the Saphyr System User Guide, following the manufacturer’s instructions (Bionano Genomics). The Saphyr chips were run to reach a minimum yield of 310 Gbp for de novo assembly. The de novo assembly and Variant Annotation Pipeline were executed on Bionano Solve software V3.7. Reporting and direct visualization of structural variants were performed on Bionano Access V1.7. The effective coverage of the assembly was about 50×, and no filtering was used. The Bionano Solve software V3.7 was used to determine the size of the repeat. While the DLE-1 enzyme does not directly label the individual repeats, the software estimates repeat lengths based on the interval between labels flanking the repeats. The minimum resolution for sizing the repeat expansions is 500 bp, which corresponds to 100 repeats for the AAGGG motif. The *RFC1* intronic AAGGG repeat size (chr4:39350045-39350103 (hg19)) was manually reviewed in the Bionano Access V1.7 genome browser.

## Results

To study the prevalence of the biallelic (AAGGG)_n_ *RFC1* repeat expansion in putative CANVAS and adult-onset ataxia patients from the Netherlands, we used the workflow illustrated in Fig. 1. Eight (89%) putative CANVAS patients as well as 59 (14.9%) and 33 (14.9%) adult-onset ataxia patients from cohorts A and B, respectively, did not have an amplifiable *RFC1* product (data not shown) and were suspected of carrying a biallelic *RFC1*  expansion, but with a yet unknown sequence motif. In those cases, we performed RP–PCR to define the nature of the repeat expansions (I, II or III, as described in Materials and Methods). The RP–PCR analysis revealed five putative CANVAS and twelve adult-onset ataxia cases (cohorts A and B) to be suspected carriers of the biallelic expanded (AAGGG)_n_ repeat (III) (Fig. 1). The other patients with a non-amplifiable *RFC1* allele and a negative RP–PCR for the (AAGGG)_n_ repeat expansion carried the expanded benign allele (AAAAG)_15–200_ or (AAAGG)_40–1000_. However, studies have shown that even with a positive RP–PCR for the (AAGGG)_n_ repeat expansion, other sequence motifs might also be present at this locus [5]. Thus, follow-up needed to be performed with Sanger sequencing of the first and last 150 base pairs of the repeat to confirm the sequence of the motif. Sanger sequencing confirmed the presence of the AAGGG motif on both expanded *RFC1* alleles in all putative CANVAS patients, while the AAGGG motif was detected in both expanded alleles in ten adult-onset ataxia patients (Fig. 1). Two patients (patients 10 and 11) from adult-onset ataxia cohort A carried a putative mono-allelic (GAAGG)_n_ repeat in combination with the mono-allelic (AAGGG)_n_ repeat (Table 1).

**Table 1:** Clinical features of patients with expanded *RFC1* alleles

Cohort	Number	Sex	AaO	F/S	Repeat sequence	Repeat size (bp)	Number of repeats	Progression ataxia	Clinical signs of neuropathy	Nerve conduction studies abnormal	MRI scan	Cerebellar syndrome	Dysautonomia	Bilateral vestibular impairment	Cough	Full-blown CANVAS
Historically collected putative CANVAS	1	M	30	F	AAGGG (biallelic)	6498/5880	1299/1176	Slowly	Yes	Yes (S)	Mild cerebellar atrophy	Yes	–	–	Yes	–
	2	F	64	F	AAGGG (biallelic)	4003/4003	800/800	Slowly	Yes	Yes (S)	Mild cerebellar atrophy	Yes	–	Yes	–	Yes
	3	F	65	F	AAGGG (biallelic)	4820/4820	964/964	Slowly	Yes	No	Mild cerebellar atrophy	Yes	No	No	No	No
	4	F	> 50	F	AAGGG (biallelic)	4087/4087	817/817	Slowly	Yes	–	No major abnormalities	Yes	Yes	Yes	Yes	Yes
	5	F	> 60	F	AAGGG (biallelic)	4281/4281	856/856	Slowly	Yes	–	Mild cerebellar atrophy	Yes	No	Yes	–	Yes
Adult-onset ataxia cohort A	6	F	47	S	AAGGG (biallelic)	4750/4750	950/950	Slowly	Yes	Yes (S)	No major abnormalities	Yes	No	Yes	Yes	Yes
	7	M	55	S	AAGGG (biallelic)	4451/4451	890/890	Slowly	Yes	Yes (S/M)	No major abnormalities	Yes	–	No	Yes	No
	8	M	70	F	AAGGG (biallelic)	–	–	Slowly	Yes	Yes (S)	Mild cerebellar atrophy	Yes	–	–	–	–
	9	M	60	F	AAGGG (biallelic)	–	–	Slowly	–	–	–	Yes	–	–	–	–
	10	M	45	S	Allele 1: AAGGG Allele 2: GAAGG	–	–	Slowly	Yes	Yes (S/M)	Mild cerebellar atrophy	Yes	No	No	–	No
	11	F	–	–	Allele 1: AAGGG Allele 2: GAAGG	–	–	–	–	–	–	Yes	–	–	–	–
Adult-onset ataxia cohort B	12	F	43	S	AAGGG (biallelic)	–	–	Slowly	Yes	Yes (S)	No major abnormalities	Yes	No	–	–	–
	13	M	53	F	AAGGG (biallelic)	–	–	Slowly	Yes	–	Cerebellar atrophy	Yes	No	–	Yes	–
	14	M	48	S	AAGGG (biallelic)	–	–	Slowly	Yes	Yes (S)	Cerebellar atrophy	Yes	No	–	Yes	–
	15	F	-	-	AAGGG (biallelic)	–	–	–	Yes	–	–	–	–	–	–	–
	16	M	-	-	AAGGG (biallelic)	–	–	–	Yes	–	–	–	–	–	–	–
	17	M	63	F	AAGGG (biallelic)	–	–	Slowly	Yes	–	Cerebral and cerebellar atrophy	Yes	Yes	Yes	–	Yes

No fresh blood samples could be obtained for patients 8–17 to determine the size of the repeat with the optical mapping technique

– unknown/data not available, AaO: age at onset, F: familial, S: sporadic, (S): Sensory, (M): Motor

To determine the length of the expanded *RFC1* repeats, we performed optical genome mapping (Bionano Genomics) for five putative CANVAS cases and two adult-onset ataxia cases from cohort A (Fig. 1 and Table 1). Optical genome mapping confirmed the presence of expanded *RFC1* alleles in all 7 cases. Overall, the *RFC1* repeat size ranged from 800 to 1299 AAGGG repeats (Table 1). Notably, six cases showed expanded biallelic (AAGGG)_n_ alleles of similar size, but this may reflect a technical limitation of the optical genome mapping technology.

### Clinical features of patients with expanded *RFC1* repeats

To assess the presence of the main clinical features of CANVAS in the 17 patients with expanded (AAGGG)_n_ repeats, we retrospectively collected the clinical data, but unfortunately not for every patient all the required information was available (Table 1).

Two of the cases (numbers 1 and 3) from our historically collected, putative CANVAS cohort did not present with overt bilateral vestibulopathy, in addition to cerebellar ataxia and neuropathy, and as such did not fulfill all the required diagnostic criteria of CANVAS [7]. Likewise, two adult-onset ataxia cases (numbers 6 and 17) had cerebellar ataxia with neuropathy and bilateral vestibular impairment but were not previously labelled as CANVAS. For the remaining cases, mostly presented with a slowly progressive cerebellar syndrome and a sensory axonal neuropathy. However, two cases (numbers 7 and 10) presented with a sensorimotor axonal neuropathy. Cough was reported in some cases and cerebellar atrophy on MRI was quite prevalently seen. The unifying clinical picture linked to *RFC1* repeat expansions in our study is an adult-onset slowly progressive cerebellar ataxia combined with a mostly sensory neuropathy, as well as bilateral vestibular impairment in some cases.

## Discussion

This is the first study to examine the *RFC1* intronic repeat region in a historically collected putative Dutch CANVAS and two adult-onset ataxia cohorts and use optical genome mapping (Bionano Genomics) to determine the size of the expanded *RFC1* repeat length. We found the previously reported biallelic (AAGGG)_n_ repeat expansion in five of the nine putative CANVAS patients (55%) and in 10 of the 617 adult-onset ataxia patients (1.6%, cohorts A + B). Five patients with biallelic (AAGGG)_n_ repeat expansions were retrospectively clinically diagnosed with full-blown CANVAS based on the presence of cerebellar ataxia, bilateral vestibulopathy, and sensory neuropathy, with the note that data on vestibular investigations were not available for many cases with an expanded *RFC1* repeat. Therefore, the number of cases with CANVAS diagnosis could be actually higher. This retrospective study also included genetic diagnostic requests from other centers which has led to missing detailed phenotypic data. Consequently, two cases from the small, historic series of putative CANVAS patients did not fulfil all diagnostic criteria for CANVAS, and two CANVAS diagnoses were not labelled as such in the adult-onset ataxia cohorts. Additionally, two patients with an expanded *RFC1* repeat presented with a sensorimotor neuropathy that was also recently described by others who reported *RFC1* repeat expansions as the cause of sensorimotor neuropathy in 3/138 cases [13]. In contrast, a large study by Curro et al., identified *RFC1* repeat expansions only in sensory neuropathy cases and not in their cohort of 100 cases with sensorimotor neuropathy [14]. Nevertheless, our work and that of others showed that the clinical spectrum associated with *RFC1* repeat expansions is broader than previously reported and patients may present with a mixed sensorimotor axonal neuropathy.

The biallelic (AAGGG)_n_ expansion was the repeat configuration found in all putative CANVAS patients and in most adult-onset ataxia cases. However, in 2 of the 12 adult-onset ataxia cases with a suspected biallelic (AAGGG)_n_ repeat expansion, we also observed a putative mono-allelic (GAAGG)_n_ repeat expansion in combination with the mono-allelic expanded (AAGGG)_n_ repeat. The size of the (AAGGG)_n_ repeat expansion ranged from 4 to 7 kb corresponding to 800–1299 pentanucleotide repeats, consistent with the previously reported repeat range.

Our diagnostic yield (55%) for the biallelic (AAGGG)_n_ repeat expansion in the small series of putative CANVAS patients is quite similar to that reported in previous studies, where biallelic (AAGGG)_n_ repeat expansions were reported to lead to a genetic diagnosis in 68 to 100% of patients suspected to have CANVAS [4, 11]. In contrast, in our two adult-onset ataxia cohorts, the prevalence of the biallelic (AAGGG)_n_ repeat expansion in *RFC1* is in the lower range of previously reported yields (1.6% vs 0–22%) [4, 9, 15]. The variability in diagnostic yield reported for adult/late-onset ataxia is likely due to the different genetic backgrounds of the cohorts, which range from European to Asian, North and South America, and possibly to the differing inclusion criteria between the studied cohorts. Additionally, our adult-onset ataxia cohorts were not preselected on any clinical feature other than cerebellar ataxia. Also, one of our cohorts (A) was not preselected for solely sporadic cases, which may have led to a lower diagnostic yield (0.5%) compared to a reported cohort preselected for sporadic cases [4, 15]; still, the yield in cohort B, in which dominant cases had been excluded, was only slightly higher (2.7%).

In all studies, including ours, that have examined both (putative) CANVAS and adult-onset ataxia cases for the biallelic (AAGGG)_n_ repeat expansion in *RFC1*, the frequency of the biallelic (AAGGG)_n_ repeat expansion is higher in CANVAS cases than in adult-onset ataxia patients. This could be due to the different clinical manifestations of the patients from the CANVAS cohort and the adult-onset ataxia cohorts. For example, despite the fact that data on the complete clinical features of CANVAS were not available for all the patients enrolled in our study, most of the putative CANVAS patients had clinically full-blown CANVAS, whereas documented typical CANVAS was less prevalent in the adult-onset ataxia patients. The identification of biallelic (AAGGG)_n_ *RFC1* expansions in our adult-onset ataxia cohort clearly indicates the need to screen adult-onset ataxia patients for expanded *RFC1* repeats when no other genetic cause is found in other known ataxia genes, and particularly when adult-onset ataxia is combined with sensory neuropathy.

Additionally, we identified a putative mono-allelic (GAAGG)_n_ repeat expansion in combination with a mono-allelic (AAGGG)_n_ repeat expansion in two patients. Marker analysis did not show any familial relationship between these two patients (data not shown). Although, the presence of the same motifs in two independent referrals may increase the likelihood that this repeat motif is pathogenic, we should further investigate the configuration of the *RFC1* repeat in the general population and additional adult-onset ataxia cases to be able to make a conclusion. Additionally, this (GAAGG)n repeat motif may be the result of the insert of a single nucleotide G preceding the original classical repeat motif AAGGG and if this is considered, the repeat motif can be read as a pathogenic (AAGGG)n repeat expansion. In all, our results together with the other previously reported repeat motifs ((AAGAG)_n_/(AGAGG)_n_ and (ACAGG)_n_ repeat expansions [5, 7]) indicate that this repeat region can be variable and that novel disease-causing repeat configurations may be revealed over time.

In this work, we successfully used optical genome mapping [16] to replace the labor intensive and time-consuming Southern blot, which has been the “gold” standard for sizing repeat expansions up to now [17]. The number of AAGGG units found with this technique in our patients (800–1299) is consistent with the previously reported size of *RFC1* repeat units (> 400) (Supplementary Table 1). The value of optical genome mapping in sizing repeat expansions in human clinical applications has already been proven by others [18], but this technique has not yet been used for the sizing of expanded *RFC1* repeats. Furthermore, a recent study reported the use of long-read sequencing (Oxford Nanopore Technologies, Oxford, UK) to simultaneously identify, size and read the motif of the *RFC1* repeat [19]. We speculate that this technology will very likely replace the current diagnostic workflow for repeat expansion disorders in the future [20].

In conclusion, we here confirm that the *RFC1* repeat expansion explains a number of adult-onset ataxia cases who present with (incomplete) key clinical features of CANVAS. Additionally, the repeat has a dynamic nature and can have different configurations, even with a positive RP–PCR for the (AAGGG)_n_ repeat motif, and thus patients with a “positive” RP–PCR should be followed up with Sanger sequencing. To improve the current diagnostic practice, patients with genetically unexplained, slowly progressive adult-onset ataxia and sensory or sensorimotor axonal neuropathy should be screened for the presence of (AAGGG)_n_ repeat expansions in the *RFC1* gene*.*

## Supplementary Information

Below is the link to the electronic supplementary material.

Supplementary file1 (DOCX 39 KB)
