# Supplementary material for: Prevalence of intronic repeat expansions in RFC1 in Dutch patients with CANVAS and adult-onset ataxia
Source: J Neurol. 2022 Jul 21;269(11):6086–93. doi: 10.1007/s00415-022-11275-9 (PMC9553829; doi:10.1007/s00415-022-11275-9)
Supplement: Supplementary file 1 — Supplementary file1 (DOCX 39 KB) [file 415_2022_11275_MOESM1_ESM.docx]

**Supplementary File**

Prevalence of intronic repeat expansions in *RFC1* in Dutch patients with CANVAS and adult-onset ataxia

Fatemeh Ghorbani^1^, Jelkje de Boer-Bergsma^1^_,_ Corien C. Verschuuren-Bemelmans^1,7^, Maartje Pennings^2^, Eddy N. de Boer^1^, Berry Kremer^3,7^, Els K. Vanhoutte^4^, Jeroen J. de Vries^3,7^, Raymond van de Berg^5^, Erik-Jan Kamsteeg^2^, Cleo C. van Diemen^1†^, Helga Westers^1†^, Bart P. van de Warrenburg^6†^, Dineke S. Verbeek^1,7^*

Content: 7 Tables

**Supplementary Table 1** – Studies performed on the *RFC1* intronic repeat expansion in CANVAS and adult-onset ataxia patients.

| **Origin** | **Cohort size** | **Cohort type** | **Classical repeat**  **Frequency**  **(AAGGG)** | **Repeat length** | | **Additional remark** | **Reference** |
| --- | --- | --- | --- | --- | --- | --- | --- |
| UK, Italy, Brazil | 23 | Familial CANVAS | 23/23 (100%) | 400-1000 repeats | | – | Cortese, Andrea, et al. (2019) |
| UK | 150 | Sporadic late-onset ataxia | 33/150 (22%) |  |  |  |  |
| Brazil | 23 | Adult-onset ataxia | 2/23(8.7%) | – | | – | Akçimen, Fulya, et al. (2019) |
| Canada | 154 | Adult-onset ataxia | 1/154 (0.6%) | – | | Novel AAGAG and AGAGG in heterozygous states |  |
| New Zealand – Māori | 13 | CANVAS | 13/13 (100%)  (embedded in benign variant (AAAGG)) | 990–1940 repeats | | (AAAGG)10–25 (AAGGG)exp | Beecroft, Sarah J., et al. (2020) |
| Japan | 37 | Familial and sporadic late-onset ataxia | 3/49 (6.1%) | 1600–2200 repeats | | Novel biallelic ACAGG repeat expansion | Tsuchiya, Mai, et al. (2020) |
| North America | 911 | Adult-onset ataxia | 29/911  (3.2%) | _ | | _ | Syriani, Dona Aboud, et al. (2020) |
| Chinese | 91 | Sporadic late-onset ataxia | 0/91(0%) | _ | | _ | Fan, Yu, et al. (2020) |
| UK, New Zealand, France, Italy, Brazil, Slovenia Australia | 70 | CANVAS | 63/70 (90%) | 433–2750 repeats | |  | Cortese, Andrea, et al. (2020) |
|  | 293 | Adult-onset ataxia | 42/293 (14%) |  |  |  |  |
| Europe (France (13), Germany (45), Italy (2), Netherlands (2), Sweden (1), Spain (9), Switzerland (4)), Turkey | 76 | CANVAS and ataxia with chronic cough | 52/76 (68.4%) | > 1880 repeats | | – | Traschütz, Andreas, et al. (2021) |
|  | 105 | Late-onset ataxia | 18/105 (17.1%) |  |  |  |  |
| Spain | 13 | CANVAS | 11/13 (85%) | _ | | _ | Costales, María, et al. (2021) |
| Italy | 40 | Chronic Idiopathic Axonal Polyneuropathy (CIAP)-pure sensory neuropathy | 21/40 (53%) | _ | | _ | Tagliapietra, Matteo, et al. (2021) |
|  | 56 | Chronic Idiopathic Axonal Polyneuropathy (CIAP)- predominantly sensory | 10/56 (18%) | _ | | _ |  |
|  | 138 | Chronic Idiopathic Axonal Polyneuropathy (CIAP)- sensorimotor | 3/138 (2%) | _ | | _ |  |
| France | 100 | idiopathic sporadic late-onset ataxia (ILOA) | 15/100 (15%) | 32- >1300 repeats | | _ | Montaut, Solveig, et al. (2021) |
|  | 21 | idiopathic early-onset ataxia (IEOA) | 0/21 (0%) | _ | | _ |  |
|  | 42 | Multiple System Atrophy of Cerebellar type (MSA-C) | 0/42 (0%) | _ | | _ |  |
| UK and Italy | 125 | Chronic idiopathic axonal polyneuropathy (sensory  neuropathy) | 43/125 (34%) | 249-2386 repeats | _ | | Curro, Riccardo, et al.(2021) |
|  | 100 | Chronic idiopathic axonal polyneuropathy (sensory-motor neuropathy) | 0/100 (0%) | _ | _ | |  |

**Supplementary Table 2** – Flanking PCR primers for the *RFC1*, *DCHS1* and *AMELX/Y* loci

| **Name** | **Sequence (5’🡪 3’)** | **PCR fragment size** |
| --- | --- | --- |
| \| *RFC1*-F \|  \| \| --- \| --- \| | TCAAGTGATACTCCAGCTACACCGTTGC | 348bp |
| *RFC1*-R | GTGGGAGACAGGCCAATCACTTCAG |  |
| *DCHS1*-F | AGCCTGGACTTGCAGATTG | 600bp |
| *DCHS1*-R | TCCAGCTGTAGCATATAGTG |  |
| *AMELX/Y-F*  *AMELX/Y-R* | CCCTGGGCTCTGTAAAGAATAGTG  ATCAGAGCTTAAACTGGGAAGCTG | 106/ 112bp |

**Supplementary Table 3** – Primers used for Repeat Primed-PCR targeting three different expanded repeats

| **Name** | **Sequence (5’🡪 3’)** |
| --- | --- |
| *RFC1*_Anchor | Tacgcatcccagtttgagacg |
| *RFC1*_F_Universal | TCAAGTGATACTCCAGCTACACCGT |
| *RFC1*_R_AAAAG | TacgcatcccagtttgagacgGAAAAGAAAAGAAAAGAAAAGAAAA |
| *RFC1*_R_AAAGG | TacgcatcccagtttgagacgGGAAAGGAAAGGAAAGGAAAGGAAA |
| *RFC1*_R_AAGGG | TacgcatcccagtttgagacgGGGAAGGGAAGGGAAGGGAA |

**Supplementary Table 4** – PCR conditions used for the Repeat Primed-PCR. Per patient, a PCR reaction for the AAAAG, AAAGG and AAGGG allele was performed.

| **Component** | **Concentration (in pmol/ µl)** | **Amount (in µl)** |
| --- | --- | --- |
| Amplitaq Gold 360 master mix | – | 12.5 |
| GC-enhancer | – | 2.5 |
| Forward primer (universal) | 10 | 1 |
| Anchor (universal) | 10 | 1 |
| Reverse primer (differs per allele) | 10 | 0.25 |
| Patient DNA (100ng) | – | – |
| MilliQ | – | – |

Total volume 25ul

**Supplementary Table 5** – PCR program used for the Repeat Primed-PCR

| **Temp (ºC)** | **Time** | **Cycles** |
| --- | --- | --- |
| 95 | 10 min |  |
| 95 | 30 sec | 35 |
| 62 | 30 sec |  |
| 72 | 60 sec |  |
| 72 | 7 min |  |
| 10 | ∞ |  |

**Supplementary Table 6** – Long range PCR conditions to generate template for Sanger sequencing of the expanded *RFC1* repeat

| **Component** | **Amount in µl** |
| --- | --- |
| gDNA (50ng) | – |
| Phusion Flash HF MM | 10 |
| *RFC1*F+R (2x0.5µM) | 2 |
| DMSO | 0.6 |
| MilliQ | – |

Total volume 20ul

**Supplementary Table 7** – Long range PCR program to generate template for Sanger sequencing of the expanded *RFC1* repeat

| **Temp (ºC)** | **Time** | **Additional setting** | **Cycle** |
| --- | --- | --- | --- |
| 98 | 3 min |  |  |
| 98 | 10 sec |  | 18 |
| 65 | 15 sec | Decrease 0.5 ºC each cycle |  |
| 72 | 3 min |  |  |
| 98 | 10 sec |  | 18 |
| 57 | 15 sec | Decrease 0.5 ºC each cycle |  |
| 72 | 3 min |  |  |
| 72 | 5 min |  |  |
| 15 | ∞ |  |  |
